# Group-specific Quantification of *mcrA* genes of Methanogenic Archaea and “*Candidatus* Methanoperedens” by Digital PCR

**DOI:** 10.1264/jsme2.ME24097

**Published:** 2025-05-09

**Authors:** Takeshi Watanabe, Atsuya Endo, Rio Hamada, Rina Shinjo, Susumu Asakawa

**Affiliations:** 1 Graduate School of Bioagricultural Sciences, Nagoya University, Furocho, Chikusa, Nagoya 464–8601, Japan; 2 School of Agricultural Sciences, Nagoya University, Furocho, Chikusa, Nagoya 464–8601, Japan

**Keywords:** methanogenic archaea, *mcrA* genes, digital PCR, paddy field soil

## Abstract

Digital PCR is a technique that quantifies target genes based on the absence or presence of the targets in PCR amplicons. The present study exami­ned group-specific probes for the quantification of *mcrA* genes in six methanogenic archaeal groups and “*Candidatus* Methanoperedens” by digital PCR with the universal primers ML-f and ML-r. A digital PCR ana­lysis of paddy field soil detected all the targets, with the dominant and minor groups being *Methanomicrobiales* and *Methanobrevibacter* spp., respectively (10^7^ and 10^4^ copies [g dry soil]^–1^). This method has the potential to reveal the dynamics of specific methanogenic archaeal groups in the environment.

Methanogenic archaea are obligate anaerobes and the only microorganisms that obtain energy by producing methane. They all belong to Archaea and are classified into‍ ‍the following eight orders: *Methanobacteriales*, *Methanococcales*, *Methanomicrobiales*, *Methanosarcinales*, *Methanopyrales*, *Methanocellales*, *Methanomassiliicoccales*, and *Methanonatronarchaeales*, in the phylum *Methanobacteriota* (Parte *et al.*, 2020). Several Candidatus taxa, including “*Candidatus* Methanomethylicales” (Vanwonterghem *et al.*, 2016), “*Candidatus* Methanodesulfokora washburnenis” (McKay *et al.*, 2019), and “*Candidatus* Methanosuratincolales” (Wu *et al.*, 2024), in the phylum *Thermoproteota* have also been proposed. Since methanogenic archaea generally utilize a limited range of substrates, typically H_2_ and CO_2_, CO, formate, and methyl compounds, including acetate, methanol, and methylamines, they are key players in the terminal decomposition processes of organic matter in anaerobic environments.

Methane is an important energy resource as the main component of natural gas, and is also the second most important greenhouse gas after CO_2_. Since its concentration in the atmosphere has continued to increase, reaching 1,932‍ ‍ppb in April 2024 (Lan *et al.*, 2022), efforts to reduce methane emissions from a number of sources, particularly by anthropogenic activities, are an urgent agenda to achieve the goals of the Global Methane Pledge. Paddy fields are one of the main anthropogenic emission sources of methane, and 86% of fields are distributed throughout Asia (FAO, 2024). To obtain insights into the ecology of methanogenic archaea in paddy fields, the diversity and dynamics of the methanogenic archaeal community in this ecosystem have been intensively investigated using not only the culture-dependent most-probable number method, but also culture-independent techniques, such as clone libraries, T-RFLP, PCR-DGGE, real-time PCR, and amplicon sequencing ana­lyses of 16S rRNA and *mcrA* genes, encoding the alpha-subunit of methyl-coenzyme M reductase (Conrad, 2007; Alpana *et al.*, 2017; Kharitonov *et al.*, 2021; Asakawa, 2021). These studies provided fundamental information on the phylogenetic composition, abundance, and dynamics of the methanogenic archaeal community in the paddy field ecosystem. However, a quantitative ana­lysis of phylogenetic group-specific methanogenic archaea in paddy field soil has yet to be conducted even though several primers and probes have been designed to detect group-specific methanogenic archaeal 16S rRNA and *mcrA* genes (*e.g.*, Narihiro and Sekiguchi, 2017; Mathai *et al.*, 2019). This may be attributed to the technical limitations and labor-intensive nature of this work. Nevertheless, quantitative data on the abundance of specific methanogenic archaeal groups are important for understanding their community dynamics.

Digital PCR is one of the gene quantification techniques that allows the copy number of a target gene to be estimated based on the absence or presence of amplified gene fragments in several thousands of nanoscale droplets or compartments with the Poisson law (Quan *et al.*, 2018). The values obtained are independent of reference curves, and the impact of PCR efficiency is relatively low; however, the dynamic range for quantification by digital PCR is generally narrower than that of a real-time PCR ana­lysis. Therefore, this technique is expected to be another useful tool for tracing the dynamics of the methanogenic archaeal community in the environment, particularly when combining universal primers with probes that detect specific groups of methanogenic archaea. More options for the quantification of methanogenic archaea will provide more detailed insights into methanogenesis in the paddy field ecosystem, such as the interactions within/between a methanogenic archaeal community, other microorganisms, and physicochemical properties. In our previous study, *pmoA* genes, encoding the beta-subunit of particulate methane monooxygenase, of type I and type II methane-oxidizing bacteria (MOB) in paddy field soil were quantified using a probe-based assay on a chip-type digital PCR platform, which contained a large number of nanoscale wells (Shinjo *et al.*, 2023). The findings obtained showed that the abundance of type I MOB‍ ‍was higher than that of type II MOB at the surface-oxic layer of flooded paddy field soil. Harada *et al.* (2024)‍ ‍applied chip-type digital PCR to the quantification of‍ ‍the *mcrA* genes of *Methanobacterium* spp. and *Methanobrevibacter* spp. living in tree trunks, in which the primer set of ME1 (or ME1-mod; Harada *et al.*, 2024) and ME2 (Hales *et al.*, 1996) was used for PCR amplification, which amplified *ca.* 720–740 bp of *mcrA* sequences. In the present study, we exami­ned digital PCR conditions using another universal *mcrA* primer set, ML-f and ML-r (Luton *et al.*, 2002) because the short lengths of the target region (*ca.* 410–435 bp) were preferable for the ana­lysis. Group-specific probes were exami­ned for the detection of the *mcrA* genes of six methanogenic archaeal members and “*Ca.* Methanoperedens”, an anaerobic methanotrophic archaeal group (Haroon *et al.*, 2013), in paddy field soil, including a re-examination of the probes for *Methanobacterium* spp. and *Methanobrevibacter* spp.

We assessed the applicability of several primers and probes designed for methanogenic archaea (Narihiro and Sekiguchi, 2017) as probes for digital PCR at the beginning of the study. However, sequence mismatches to targets and/or lower *T*_m_ values were not appropriate for the ana­lysis of methanogenic archaeal groups in paddy fields. Therefore, group-specific probes were newly designed. In total, 385 *mcrA* nucleotide sequences of methanogenic archaeal isolates were obtained from the FunGene database (Fish *et al.*, 2013) on September 30, 2021, and were aligned by Clustal W (Thompson *et al.*, 1994), as described by Harada *et al.* (2024). Candidate probe annealing sites, which were conserved in each target group, were searched manually between the primer regions of ML-f and ML-r. Sequence variations between the candidate sites and *mcrA* clones obtained from paddy field soil using the primer set ML-f and ML-r (Watanabe *et al.*, 2009) were also manually checked after alignments. Twenty-nine candidate probes were designed for the detection of *Methanobacterium* spp.,‍ ‍*Methanobrevibacter* spp., *Methanosarcina* spp., *Methanothrix* spp., *Methanocella* spp., and members of *Methanomicrobiales* other than *Methanocorpusculum*, which were the major methanogenic archaeal groups in paddy field soil (Asakawa, 2021). We did not target *Methanocorpusculum* spp. in the present study because of‍ ‍marked differences in their *mcrA* sequences from those in‍ ‍the other groups of *Methanomicrobiales* and no *Methanocorpusculum* spp. being isolated from the paddy field environment. The primer McrA1360R, which was designed for the ana­lysis of “*Ca.* Methanoperedens” (Vaksmaa *et al.*, 2017), was also exami­ned as a candidate probe. All probes were designed to hybridize to the sense strand of target DNA and were synthesized by Integrated DNA Technologies. The 5′-terminal nucleotide was modified with the fluorophore 6-carboxyfluorescein (6-FAM) or Yamaki yellow, while the internal and 3′-terminal nucleotides were modified with ZEN and Iowa Black FQ, respectively, as quenchers.

The genomic DNA and/or *mcrA* amplicons of 20 methanogenic archaeal strains and 24 representative *mcrA* clones obtained from paddy field soil (Watanabe *et al.*, 2009) were used as template DNA to examine the validity of the candidate probes for the digital PCR assay (Table S1). Their copy numbers were adjusted to *ca.* 10^3^ copies μL^–1^. In the present study, the QIAcuity digital PCR System (Qiagen) and QIAcuity nanoplates (8.5 k), which contain 8,500 partitions in each well, were used for assays. Fifteen microliters of the reaction mixture contained 3.75‍ ‍μL of 4× Probe PCR Master Mix, 0.24‍ ‍μL each of the forward (ML-f) and reverse (ML-r) primers (50‍ ‍μM each), 0.15‍ ‍μL of the probe (10‍ ‍μM), 0.15‍ ‍μL of bovine serum albumin (20‍ ‍mg mL^–1^), 2.5‍ ‍μL of the DNA template, and 7.97‍ ‍μL of sterilized ultrapure water, in which 12‍ ‍μL of the mixture was loaded onto each well. Two-step PCR was performed as follows: initial denaturation at 95°C for 2‍ ‍min, 40 cycles of denaturation at 95°C for 30‍ ‍s, and annealing and elongation at a given temperature for 3‍ ‍min.

Annealing and elongation temperatures of 60°C were initially exami­ned. However, some of the probes did not work well, namely, target groups were not detected even if the probe sequences matched the target sequences and/or non-targets were detected even if there were some mismatches to the probe sequences (data not shown). Since *T*_m_ values differed among the candidate probes, PCR conditions were optimized by examining different annealing and elongation temperatures for each candidate probe to detect targets with a distinct fluorescence intensity. We then fixed the PCR conditions for all target groups with the primer set ML-f/ML-r (Table 1). All the probes detected the targeted *mcrA* genes with a distinct fluorescence intensity; however, some targets showed lower numbers than other targets (Table S2 and Fig. S1). Since the number of mismatches between the probes and most of the target *mcrA* sequences was 0 or 1, the low number of positive counts in some cases appeared to be attributed to the low affinity of primer annealing. Furthermore, some probes detected non-targeted *mcrA* genes; however, the number of positive counts was two orders of magnitude lower than the expected values (*e.g.*, D-FL-15 with MCR_Mthx and D-FL-4 and R-FL-18 with MCR_Mmic in Table S2 and Fig. S1) or the fluorescence intensity was low and was distinguished from those of the targets (“low signal” in Table S2 and Fig. S1). Therefore, these effects were considered to be negligible. The present study exami­ned a number of probes for *mrtA* of *Methanobacterium* spp. and *Methanobrevibacter* spp., an isozyme of *mcrA*, because some *mrtA* clones were previously retrieved from paddy field soil samples (Watanabe *et al.*, 2009). However, their detection ranges, particularly for *Methanobrevibacter* spp., were not sufficient. Although this may have been due to primer mismatches, sequence availability was also limited and the presence of *mrtA* was not confirmed in some strains (Table S2). Therefore, caution is needed regarding the quantification of *mrtA*.

The precision of digital PCR quantification was confirmed by examining the genomic DNA templates of *Methanobacterium palustre* F^T^ (Zellner *et al.*, 1988), *Methanobrevibacter arboriphilus* SA (Asakawa *et al.*, 1993), *Methanosarcina mazei* TMA (Asakawa *et al.*, 1995), and *Methanoculleus chikugoensis* MG62^T^ (Dianou *et al.*, 2001), which were serially diluted by a factor of 2 from 10^3^ or 10^4^ to 10^1^ or 10^2^ copies μL^–1^. Two-fold differences in *mcrA* gene copies were precisely detected for all targets with small variations (slope≈1.0, R^2^>0.998, coefficient variation <6.3%), except for 10^1^ copies μL^–1^ when the number of PCR-positive partitions in a well (8,500 partitions) was very low (Fig. 1). These results show that optimized digital PCR conditions are useful for showing slight differences in the abundance of target methanogenic archaeal groups.

To examine the applicability of PCR conditions to paddy environments, DNA samples extracted from soil collected from three paddy fields were subjected to digital PCR assays. One paddy field soil sample was obtained from a double-cropping paddy field (rice-wheat) in the Aichi Agricultural Research Center (Anjo; latitude 34°58′20″N, longitude 137°04′28″E) on September 2, 2015. The two other paddy field soil samples were collected from plots for the long-term experimental field of chemical fertilizers (NPK plot) and rice straw compost (RSC plot) in the NARO Tohoku Agricultural Research Center (Omagari; latitude 39°29′28″N, longitude 140°29′47″E) on August 12, 2015. Details on field management and DNA extraction procedures were previously described by Naruse *et al.* (2019).

Digital PCR assays using the exami­ned probes successfully detected all target groups (Fig. 2 and S1). In all three soil samples, members of *Methanomicrobiales* and *Methanothrix* spp. were the most abundant and second most abundant groups, with copy numbers of 1.6×10^7^–2.7×10^7^ and 8.0×10^6^–1.6×10^7^ copies (g dry soil)^–1^, respectively. *Methanocella* spp., *Methanosarcina* spp., and *Methanobacterium* spp. were the next most abundant group at 2.8×10^6^–5.0×10^6^ copies (g dry soil)^–1^. The copy numbers of *Methanobrevibacter* spp. were <7.2×10^5^ copies (g dry soil)^–1^, but were successfully detected in all soil samples. “*Ca.* Methanoperedens” spp. were detected with large variations; copy numbers in Anjo paddy field soil and Omagari paddy field soil were 7.4×10^6^ and <1.5×10^6^ copies (g dry soil)^–1^, respectively. The copy number of total *mcrA* genes estimated by real-time PCR with the same primer set, ML-f/ML-r was *ca.* 10^7^ copies (g dry soil)^–1^ (Fig. 2). The ratio of the cumulative number of *mcrA* genes by digital PCR to total *mcrA* genes by real-time PCR was 0.78–1.11. Furthermore, there was a correlation between them (*r*=0.79, *P*<0.05). Since the primer set ML-f and ML-r was used in both digital PCR and real-time PCR assays, the high recovery ratio and correlation observed suggested that each probe hybridized well with the targets in the DNA extracts derived from paddy field soil.

In comparisons of the relative composition of the methanogenic archaeal community in the three paddy field soil samples, marked differences were observed between the communities in the Anjo and Omagari paddy fields; the relative abundances of *Methanomicrobiales* and *Methanothrix* spp. were higher in the NPK and RSC plots in the Omagari paddy field (Tukey’s HSD test, *P*<0.05 and *P*<0.052, respectively), while those of “*Ca.* Methanoperedens” spp. and *Methanobrevibacter* spp. were higher in Anjo soil (both *P*<0.01). Although no marked differences were observed between the NPK and RSC plots in the Omagari paddy field, the sum of the copy numbers was slightly higher in the RSC plot than in the NPK plot. These results are consistent with previous findings obtained from a DGGE ana­lysis (Watanabe *et al.*, 2006, 2007); site differences markedly affected the community structure of methanogenic archaea in paddy field soil, while the effect of organic fertilizer management on the community structure, particularly on its composition, was negligible in the same site.

Therefore, the digital PCR method with the designed probes is useful for quantifying the abundance of target methanogenic archaeal groups in paddy field soil and tracing their dynamics. It is also possible to quantitatively analyze the interactions between specific groups of methanogenic archaea and other microorganisms (*e.g.*, MOB and iron- and sulfate-reducing bacteria) and their relationship with methane dynamics. Further investigations will provide more detailed insights into the ecological roles of the methanogenic archaeal community and their contribution to methanogenesis in the paddy field ecosystem.

## Citation

Watanabe, T., Endo, A., Hamada, R., Shinjo, R., and Asakawa, S. (2025) Group-specific Quantification of *mcrA* genes of Methanogenic Archaea and “*Candidatus* Methanoperedens” by Digital PCR. *Microbes Environ ***40**: ME24097.

https://doi.org/10.1264/jsme2.ME24097

## Supplementary Material

Supplementary Material

## Figures and Tables

**Fig. 1.**
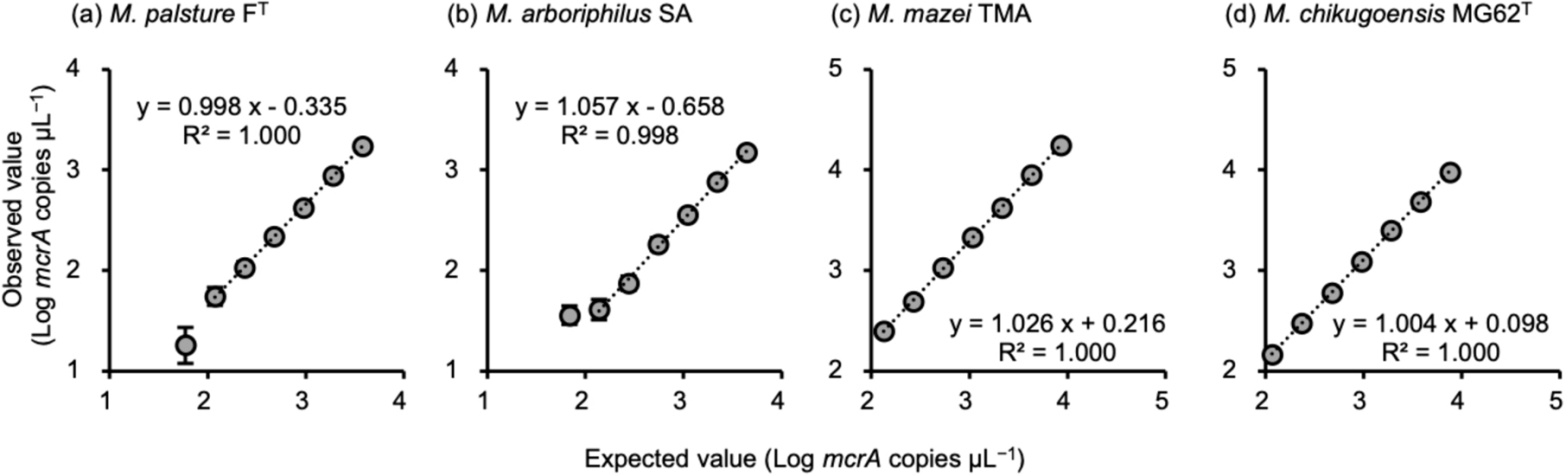
Relationship between expected and observed values of *mcrA* gene copies of four methanogenic archaeal strains. (a) *Methanobacterium palustre* F^T^, (b) *Methanobrevibacter arboriphilus* SA, (c) *Methanosarcina mazei* TMA, and (d) *Methanoculleus chikugoensis* MG62^T^. Genomic DNA templates were serially diluted by a factor of 2. The expected values were calculated from the concentration of DNA and genome length. Regression lines were drawn using all values, except for 10^1^ copies μL^–1^. Bars show the standard deviation of three measured values.

**Fig. 2.**
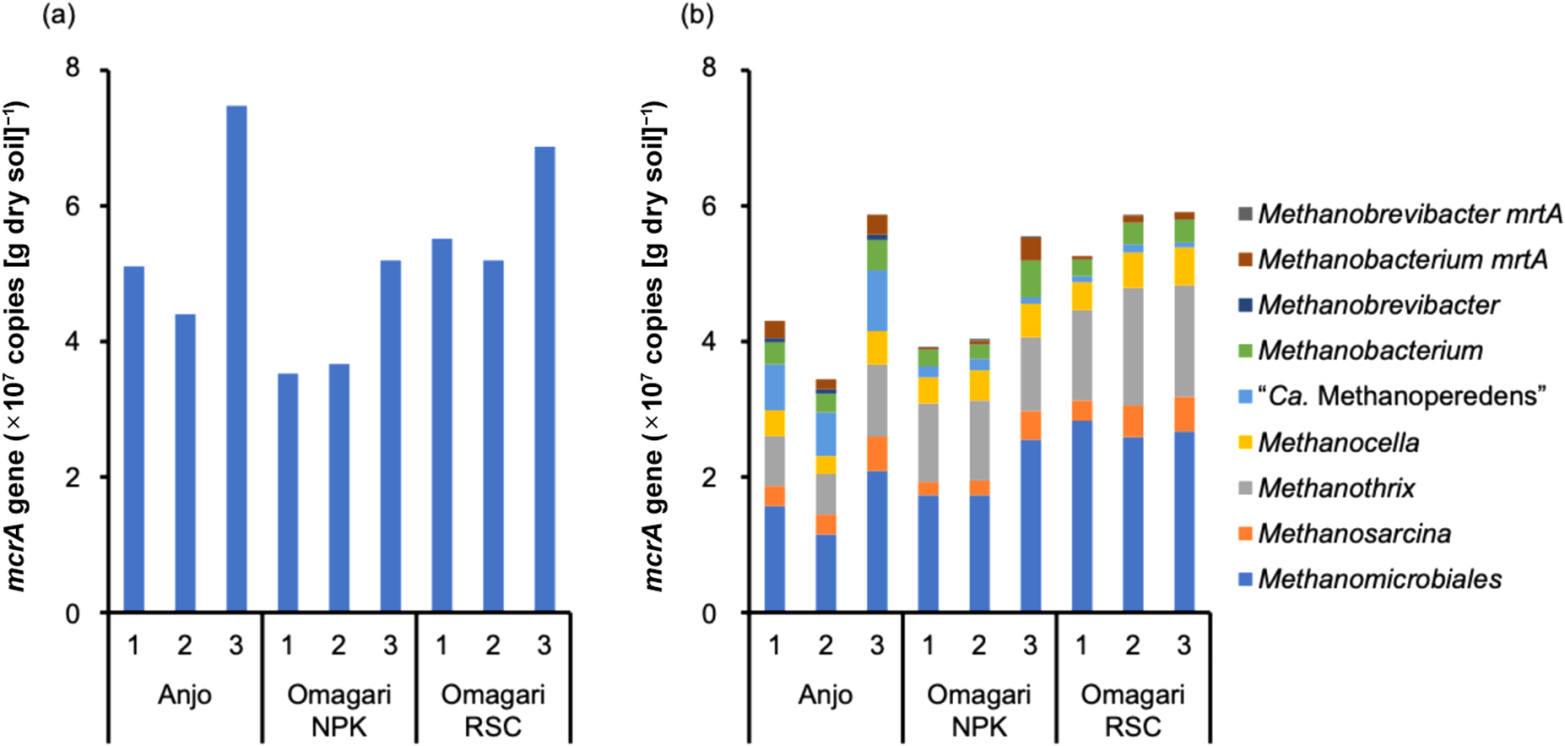
The copy number of *mcrA* genes in paddy field soil quantified by real-time PCR (a) and digital PCR (b). Anjo and Omagari NPK and RSC indicate paddy field plots in the Aichi Agricultural Research Center and NARO Tohoku Agricultural Research Center. Numbers are the replicates of DNA extraction. The primer set ML-f and ML-r (Luton *et al.*, 2002) was used in the real-time PCR ana­lysis. Details on the PCR program, reaction mixture, and reference standards were previously described (Watanabe *et al.*, 2010). The absolute values of reference standards for real-time PCR were calibrated by quantifying them using digital PCR.

**Table 1.** Group-specific probes targeting *mcrA* and *mrtA* genes of methanogenic archaea.

Target	Probe	Probe sequence (5′–3′)*	Length (mer)	Position^†^	Tm (°C)^‡^	Annealing temp. (°C)	References
*Methanobacteriales*							
*Methanobacterium* (*mcrA*)	MCR_Mbac_I^§^	TAGAAACCAAGACGTGYGTGCTGTTCTT	28	1,304–1,331	59.2–61.1	58	This study
	MCR_Mbac_II^§^	TAGAATCCGAGTCTGGAATGCTGTTCTT	28	1,304–1,331	58.3		
	MCR_Mbac_III^§^	TAGAAWCCAAGTCTGGAGYGCTGTTCTT	28	1,304–1,331	58.3–60.5		
	MCR_Mbac_IV^§^	TAGAAACCAAGTCGACTGTGCTGTTCTT	28	1,304–1,331	58.9		
	MCR_Mbac_V^§^	TAGAAACCAAGTCKGCTGTGCTGTTCTT	28	1,304–1,331	59.1–60.9		
*Methanobacterium* (*mrtA*)	MRT_Mbac	TGGCTTAAGTACCAWCCGTTRATTCCDGCGTT	32	1,261–1,292	61.8–64.9	60	This study
*Methanobrevibacter* (*mcrA*)	MCR_Mbrev	CGTTRGMWGCACCACATTGRTCTTGTAARTCG	32	1,338–1,369	58.5–64.5	57	This study
*Methanobrevibacter* (*mrtA*)	MRT_Mbrev	CCWGCATTRGARTTTCCTGTTGCAAAWGC	29	1,240–1,268	58.2–61.5	57	This study
*Methanosarcinales*							
*Methanosarcina*	MCR_Msar	TACATGGAGAGGTACCARCCWGAGAGACC	29	1,267–1,295	61.0–62.6	62	This study
*Methanothrix*	MCR_Mthx	AGSAGAAGTCRTCCAGKACRTCGTTGG	27	1,016–1,042	58.7–64.4	58	This study
*Ca.* Methanoperedens	McrA1360	TGCCTCTTTGTGGAGGTACATGGA	24	1,366–1,390	58.9	56	Vaksmaa *et al.* (2017)
*Methanocellales*							
*Methanocella*	MCR_Mcel_I^§^	ACATTGACATGTACCAGCCGGACA	24	1,269–1,294	59.5	62	This study
	MCR_Mcel_II^§^	ACATGGACAGGTACCAGGCCGACA	24	1,269–1,294	62.9		
*Methanomicrobiales* (other than *Methanocorpusculum*)	MCR_Mmic	AGAANCCGAGMCGYGACCA	19	1,312–1,330	54.2–63.3	54	This study

* All probes were designed to hybridize to the sense strand of the target DNA. ^†^ Positions were shown based on the *mcrA* sequence of *Methanothermobacter thermautotrophicus* delta H (Accession No. U10036) ^‡^ Melting temperatures (*T*_m_) were estimated using IDT OligoAnalyzer Tool (https://sg.idtdna.com/calc/analyzer), at which probe, Na^+^, and Mg^2+^ concentrations were set to 0.1‍ ‍μM, 50‍ ‍mM, and 0‍ ‍mM, respectively. ^§^ Mixtures of five probes (MCR_Mbac_I–V) and two probes (MCR_Mcel_I and II) were used for the quantification of *Methanobacterium* (*mcrA*) and *Methanocella*, respectively.
