# Supplementary material for: Group-specific Quantification of mcrA genes of Methanogenic Archaea and “Candidatus Methanoperedens” by Digital PCR
Source: Microbes Environ. 2025 May 9;40(2):ME24097. doi: 10.1264/jsme2.ME24097 (PMC12213062; doi:10.1264/jsme2.ME24097)
Supplement: Supplementary file 1 — Supplementary Material [file 40_24097_s1.pdf]

Supplementary Information

Group-specific quantification of *mcrA* genes of methanogenic archaea and "*Candidatus* Methanoperedens" by digital PCR

Takeshi Watanabe[1*], Atsuya Endo[1], Rio Hamada[2], Rina Shinjo[1], Susumu Asakawa[1]

[1]Graduate School of Bioagricultural Sciences, Nagoya University, Furocho, Chikusa, Nagoya 464-8601, Japan
[2]School of Agricultural Sciences, Nagoya University, Furocho, Chikusa, Nagoya 464-8601, Japan

Corresponding author
*Takeshi Watanabe
Tel: +81-52-789-5509, Fax: +81-52-789-4136
E-mail: watanabe@agr.nagoya-u.ac.jp

ORCID ID
Takeshi Watanabe: 0000-0001-5476-5648
Rina Shinjo: 0000-0002-3966-3560
Susumu Asakawa: 0000-0002-5878-0674

Table S1. List of methanogenic archaeal strains and *mcrA* clones used in this study.

Order	Family	Genus	Species/environmental clone	gDNA/PCR	GenBank *mcrA*	GenBank genome	Remarks
Methanobacteriales							
	Methanobacteriaceae						
		Methanobacterium					
			Methanobacterium palustre F^{T}	gDNA	AB542753		DSM 3108^{T} = NBRC 105230^{T} = OCM 238^{T}
			Methanobacterium sp. AH1 (*mcrA*)	PCR	not registered		NBRC 103406
			Methanobacterium sp. F	gDNA	not registered		NBRC 103407
			R-UP-64 (*mcrA*)	PCR	AB301403		OTU18 (Watanabe et al., 2009)
			R-UP-15 (*mcrA*)	PCR	AB301368		
			R-FL-40 (*mrtA*)	PCR	AB301336		OTU17 (Watanabe et al., 2009)
		Methanobrevibacter					
			Methanobrevibacter arboriphilus SA	gDNA	AB300777	AP019779.1	DSM 7056 = JCM 9315 = NBRC 10122 = OCM 783
			Methanobrevibacter arboriphilus A2	gDNA	not registered	GCA_027925065.1	DSM 2462 = JCM 9316
			Methanobrevibacter arboriphilus AZ	gDNA	not registered		DSM 744 = OCM 137
			Methanobrevibacter arboriphilus DC	gDNA	not registered		DSM 1536
			Methanobrevibacter arboriphilus DH1^{T}	gDNA	not registered	GCA_002072215.1	DSM 1125^{T} = ATCC 33747^{T} = JCM 13429^{T} = OCM 147^{T}
			R-UP-8 (*mrtA*)	PCR	AB301361		OTU17 (Watanabe et al., 2009)
		Methanothermobacter					
			Methanothermobacter thermautotrophicus ΔH^{T}	PCR	U10036	AE000666.1	DSM 3720^{T} = JCM 10044^{T} = NBRC 100330^{T} = OCM 143^{T} =VKM B-1908^{T}
			Methanothermobacter wolfeii DSM 2970^{T}	PCR	AB300780	CP104550.1	ATCC 43096^{T} = JCM 14652^{T} = NBRC 100332^{T} = OCM 154^{T} =VKM B-1829^{T}
Methanocellales							
	Methanocellaceae						
		Methanocella					
			D-FL-4	PCR	AB301233		OTU15 (Watanabe et al., 2009)
			R-UP-70	PCR	AB301407		OTU16 (Watanabe et al., 2009)
Methanosarcinales							
	Methanosarcinaceae						
		Methanosarcina					
			Methanosarcina mazei TMA	gDNA	AB300778	AP019780.1	DSM 9195 = JCM 9314 = NBRC 101201 = OCM 784
			Methanosarcina mazei S-6^{T}	gDNA	AB300781	CP009512.1	DSM 2053^{T} = OCM 26^{T} = VKM B-1636^{T}
			Methanosarcina mazei LYC	gDNA	AB300782	CP009513.1	DSM 4556 = ATCC 43573 = OCM 34
			D-FL-58	PCR	AB301283		OTU13 (Watanabe et al., 2009)
	Methanotrichaceae						
		Methanothrix					
			D-FL-14	PCR	AB301242		OTU14 (Watanabe et al., 2009)
			D-FL-36	PCR	AB301264		
	Candidatus Methanoperedenaceae						
		Candidatus Methanoperedence					
			D-FL-15	PCR	AB301243		OTU11 (Watanabe et al., 2009)
			R-FL-18	PCR	AB301317		OTU12 (Watanabe et al., 2009)
			D-FL-80	PCR	AB301301		
			R-FL-50	PCR	AB301344		
			D-FL-45	PCR	AB301272		

Methanomicrobiales				
Methanomicrobiaceae				
Methanoculleus				
Methanoculleus chikugoensis MG62^{T}	gDNA	AB300779	AP019781.1	DSM 13459^{T} = JCM 10825^{T} = NBRC 101202^{T} = OCM 785^{T}
Methanoculleus thermophilus CR-1^{T}	PCR	AB300783	GCA_900101055.1	DSM 2373^{T} = ATCC 33837^{T} = OCM 174^{T}
Methanoculleus palmolei INSULZ^{T}	PCR	AB300784		DSM 4273^{T}
Methanoculleus bourgensis MS2^{T}	PCR	AB300787	HE964772.2	DSM 3045^{T} = ATCC 43281^{T} = OGC 15^{T}
Methanoculleus bourgensis RC/ER	PCR	AB300785		DSM 2772 = ATCC 35293 = OCM 52
Methanoculleus bourgensis CB1	PCR	AB300786		DSM 6216
Methanoculleus marisnigri JR1^{T}	PCR	KM041259	CP000562.1	DSM 1498^{T} = ATCC 35101^{T} = OCM 56^{T}
Methanoregulaceae				
D-FL-25	PCR	AB301253		OTU1 (Watanabe et al., 2009)
D-FL-77	PCR	AB301298		OTU2 (Watanabe et al., 2009)
D-FL-2	PCR	AB301231		OTU3 (Watanabe et al., 2009)
D-FL-72	PCR	AB301294		OTU4 (Watanabe et al., 2009)
D-FL-34	PCR	AB301262		OTU5 (Watanabe et al., 2009)
D-FL-18	PCR	AB301246		OTU6 (Watanabe et al., 2009)
D-FL-54	PCR	AB301279		OTU7 (Watanabe et al., 2009)
D-FL-3	PCR	AB301232		OTU8 (Watanabe et al., 2009)
D-FL-19	PCR	AB301247		OTU9 (Watanabe et al., 2009)
R-UP-23	PCR	AB301376		OTU10 (Watanabe et al., 2009)

Table S2. Summary of the detection range of each probe by digital PCR.

Order	Family	Genus	Species/environmental clone	Template	Probe: MCR_Mbac_I-V (28 mers)		MRT_Mbac (32 mers)		MCR_Mbrev (32 mers)		MRT_Mbrev (29 mers)		MCR_Mcel_I & II (24 mers)		MCR_Msar (29 mers)		MCR_Mthx (27 mers)		McrA1360 (25 mers)		MCR_Mmic (19 mers)	
Methanobacteriales																						
	Methanobacteriaceae																					
		Methanobacterium																				
			Methanobacterium palustre F^T	gDNA	+	0	+	0	low signal	3	−	2	n.e.	9	n.e.	7	n.e.	6	n.e.	6	n.e.	9
			Methanobacterium sp. AH1	PCR	+	0	−	12	low signal	4	−	9	n.e.	8	n.e.	6	n.e.	7	n.e.	6	n.e.	9
			Methanobacterium sp. F	gDNA	+	0	±	0	low signal	4	−	3	n.e.	7	n.e.	9	n.e.	6	n.e.	6	n.e.	8
			R-UP-64 (*mcrA*)	PCR	+	0	n.e.	12	low signal	3	n.e.	9	n.e.	7	n.e.	9	n.e.	6	n.e.	6	n.e.	7
			R-UP-15 (*mcrA*)	PCR	±	3	n.e.	14	−	5	n.e.	8	n.e.	7	n.e.	9	n.e.	7	n.e.	6	n.e.	5
			R-FL-40 (*mrtA*)	PCR	n.e.	7	+	0	n.e.	4	−	5	n.e.	13	n.e.	15	n.e.	7	n.e.	13	n.e.	10
		Methanobrevibacter																				
			Methanobrevibacter arboriphilus SA	gDNA	−	4	−	14	±	0	−	6	n.e.	9	n.e.	11	n.e.	9	n.e.	6	n.e.	8
			Methanobrevibacter arboriphilus A2	gDNA	−	4	−	14	+	0	−	6	n.e.	9	n.e.	11	n.e.	9	n.e.	6	n.e.	8
			Methanobrevibacter arboriphilus AZ	gDNA	−	no data	−	no data	+	no data	−	no data	n.e.	no data	n.e.	no data	n.e.	no data	n.e.	no data	n.e.	no data
			Methanobrevibacter arboriphilus DC	gDNA	−	no data	−	no data	+	no data	−	no data	n.e.	no data	n.e.	no data	n.e.	no data	n.e.	no data	n.e.	no data
			Methanobrevibacter arboriphilus DH1^T	gDNA	−	4	−	14	+	0	−	6	n.e.	9	n.e.	11	n.e.	9	n.e.	6	n.e.	8
			R-UP-8 (*mrtA*)	PCR	n.e.	6	−	5	n.e.	7	+	0	n.e.	12	n.e.	13	n.e.	7	n.e.	11	n.e.	9
		Methanothermobacter																				
			Methanothermobacter thermautotrophicus ΔH^T	PCR	low signal	4	−	13	low signal	4	−	7	n.e.	6	n.e.	5	n.e.	6	n.e.	6	n.e.	5
			Methanothermobacter wolfeii DSM 2970^T	PCR	low signal	2	−	15	−	5	−	4	n.e.	6	n.e.	6	n.e.	8	n.e.	6	n.e.	6
Methanocellales																						
	Methanocellaceae																					
		Methanocella																				
			D-FL-4	PCR	n.e.	11	n.e.	14	n.e.	5	n.e.	8	+	1	low signal	3	−	6	−	3	±	5
			R-UP-70	PCR	n.e.	12	n.e.	14	n.e.	6	n.e.	9	+	0	−	5	−	4	−	3	n.e.	5
Methanosarcinales																						
	Methanosarcinaceae																					
		Methanosarcina																				
			Methanosarcina mazei TMA	gDNA	n.e.	10	n.e.	12	n.e.	7	n.e.	9	−	4	+	0	−	11	−	3	n.e.	5
			Methanosarcina mazei S-6^T	gDNA	n.e.	10	n.e.	13	n.e.	7	n.e.	4	−	4	+	0	−	8	−	4	n.e.	5
			Methanosarcina mazei LYC	gDNA	n.e.	10	n.e.	12	n.e.	7	n.e.	9	−	4	+	0	−	10	−	3	n.e.	5
			D-FL-58	PCR	n.e.	10	n.e.	13	n.e.	7	n.e.	8	n.e.	4	+	0	n.e.	10	−	3	n.e.	3
	Methanotrichaceae																					
		Methanothrix																				
			D-FL-14	PCR	n.e.	11	n.e.	16	n.e.	7	n.e.	12	−	8	−	11	+	0	−	10	n.e.	6
			D-FL-36	PCR	n.e.	11	n.e.	17	n.e.	8	n.e.	11	low signal	4	n.e.	8	+	1	n.e.	6	n.e.	6
	Candidatus Methanoperedenaceae																					
		Candidatus Methanoperedence																				
			D-FL-15	PCR	n.e.	6	n.e.	12	n.e.	7	n.e.	8	−	5	low signal	4	±	7	+	0	−	9
			R-FL-18	PCR	n.e.	6	n.e.	14	n.e.	7	n.e.	9	−	5	−	4	−	7	−	0	±	9
			D-FL-80	PCR	n.e.	9	n.e.	13	n.e.	6	n.e.	7	−	6	−	7	−	6	+	3	−	5
			R-FL-50	PCR	n.e.	9	n.e.	12	n.e.	8	n.e.	8	−	5	low signal	4	−	6	+	0	−	5
			D-FL-45	PCR	n.e.	10	n.e.	13	n.e.	6	n.e.	5	−	4	low signal	5	−	9	+	1	−	5

Methanomicrobiales																			
Methanomicrobiaceae																			
Methanoculleus																			
Methanoculleus chikugoensis MG62^T	gDNA	n.e.	13	n.e.	11	n.e.	11	n.e.	8	n.e.	5	n.e.	8	n.e.	6	n.e.	6	+	0
Methanoculleus thermophilus CR-1^T	PCR	n.e.	13	n.e.	11	n.e.	10	n.e.	7	n.e.	5	n.e.	7	n.e.	6	n.e.	6	+	0
Methanoculleus palmolei INSULZ^T	PCR	n.e.	12	n.e.	12	n.e.	11	n.e.	7	n.e.	7	n.e.	7	n.e.	6	n.e.	6	+	0
Methanoculleus bourgensis MS2^T	PCR	n.e.	13	n.e.	10	n.e.	10	n.e.	7	n.e.	9	n.e.	8	n.e.	6	n.e.	8	+	0
Methanoculleus bourgensis RC/ER	PCR	n.e.	13	n.e.	10	n.e.	10	n.e.	7	n.e.	9	n.e.	8	n.e.	6	n.e.	8	+	0
Methanoculleus bourgensis CB1	PCR	n.e.	12	n.e.	10	n.e.	10	n.e.	8	n.e.	9	n.e.	8	n.e.	6	n.e.	8	+	1
Methanoculleus marisnigri JR1^T	PCR	n.e.	13	n.e.	10	n.e.	10	n.e.	7	n.e.	6	n.e.	9	n.e.	6	n.e.	7	+	0
Methanoregulaceae																			
D-FL-25	PCR	n.e.	11	n.e.	11	n.e.	10	n.e.	7	n.e.	5	n.e.	10	n.e.	6	n.e.	9	+	0
D-FL-77	PCR	n.e.	11	n.e.	10	n.e.	10	n.e.	9	n.e.	5	n.e.	11	n.e.	5	n.e.	9	+	0
D-FL-2	PCR	n.e.	11	n.e.	10	n.e.	10	n.e.	8	n.e.	4	n.e.	9	n.e.	5	n.e.	8	+	0
D-FL-72	PCR	n.e.	11	n.e.	10	n.e.	10	n.e.	7	n.e.	4	n.e.	9	n.e.	6	n.e.	7	+	0
D-FL-34	PCR	n.e.	11	n.e.	11	n.e.	10	n.e.	9	n.e.	5	n.e.	10	n.e.	6	n.e.	9	+	0
D-FL-18	PCR	n.e.	12	n.e.	10	n.e.	10	n.e.	10	n.e.	6	n.e.	11	n.e.	6	n.e.	8	+	0
D-FL-54	PCR	n.e.	11	n.e.	10	n.e.	11	n.e.	8	n.e.	4	n.e.	9	n.e.	5	n.e.	6	+	0
D-FL-3	PCR	n.e.	13	n.e.	11	n.e.	11	n.e.	9	n.e.	6	n.e.	7	n.e.	4	n.e.	8	+	1
D-FL-19	PCR	n.e.	11	n.e.	11	n.e.	11	n.e.	9	n.e.	7	n.e.	9	n.e.	4	n.e.	8	+	1
R-UP-23	PCR	n.e.	11	n.e.	10	n.e.	10	n.e.	13	n.e.	6	n.e.	11	n.e.	5	n.e.	5	+	0

The red boxes indicate the *mcrA* genes targeted by each probe.
+, detected; ±, detected, but low copy numbers; −, not detected; n.e., not examined. The "low signal" indicates that some fluorescence signals were observed but the intensity was low.
The numbers in each column indicate the minimum number of mismatches between probe and *mcrA* sequences.

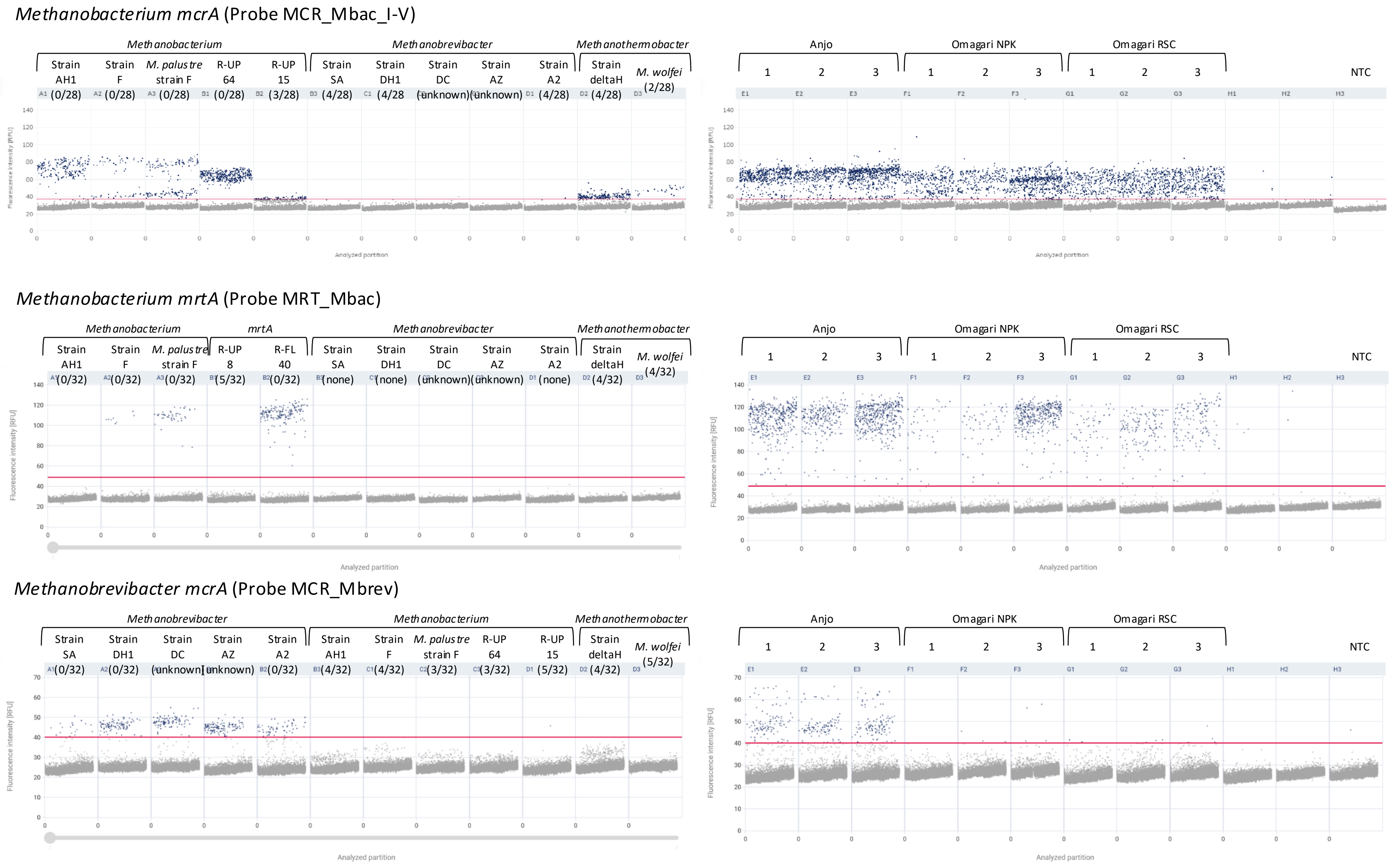

Fig. S1. Digital PCR quantification of *mcrA* genes of methanogenic archaeal groups. The sample names correspond to the list in Table S1. Anjo and Omagari NPK and RSC indicate the paddy soil samples obtained from a double-cropping paddy field (rice-wheat) in the Aichi Agricultural Research Center (Anjo) and from the plots for the long-term chemical fertilizer (NPK plot) and rice straw compost (RSC plot) examination in the NARO Tohoku Agricultural Research Center (Omagari). NTC, negative control. Threshold line (red) was manually determined. Blue and gray dots of each sample indicate positive and negative wells. The numbers in parentheses indicate the number of mismatches to probes.

Methanobrevibacter mrtA (Probe MRT_Mbrev)

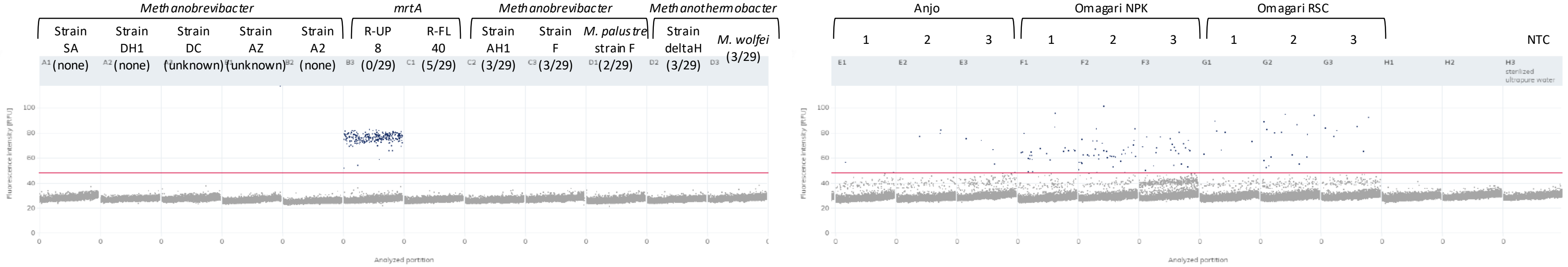

Methanosarcina (Probe MCR_Msar)

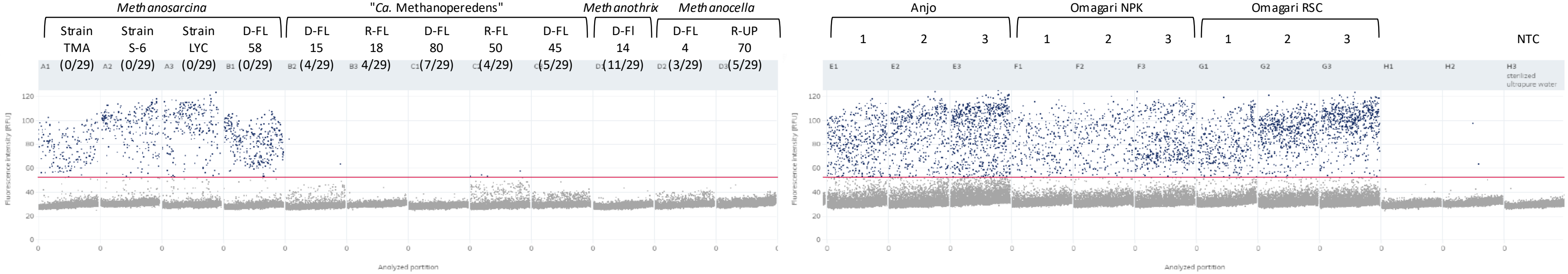

Methanothrix (Probe MCR_Mthx)

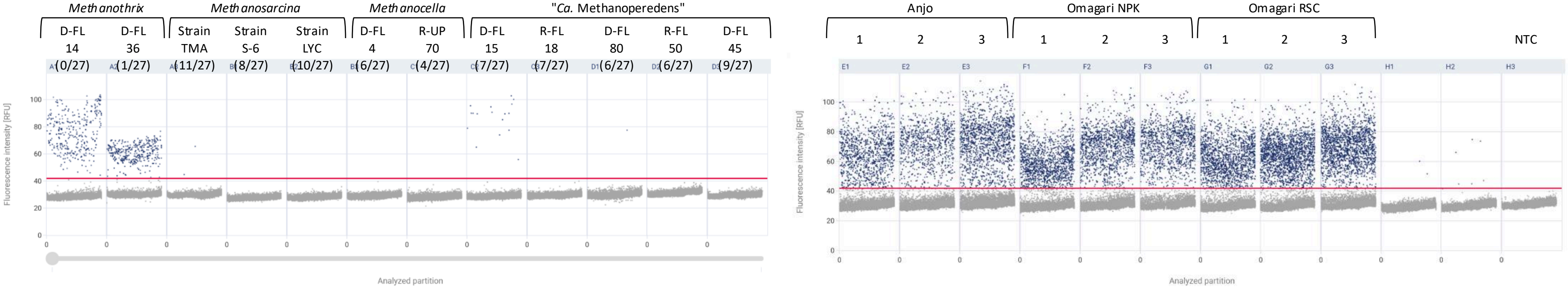

Fig. S1. Continued.

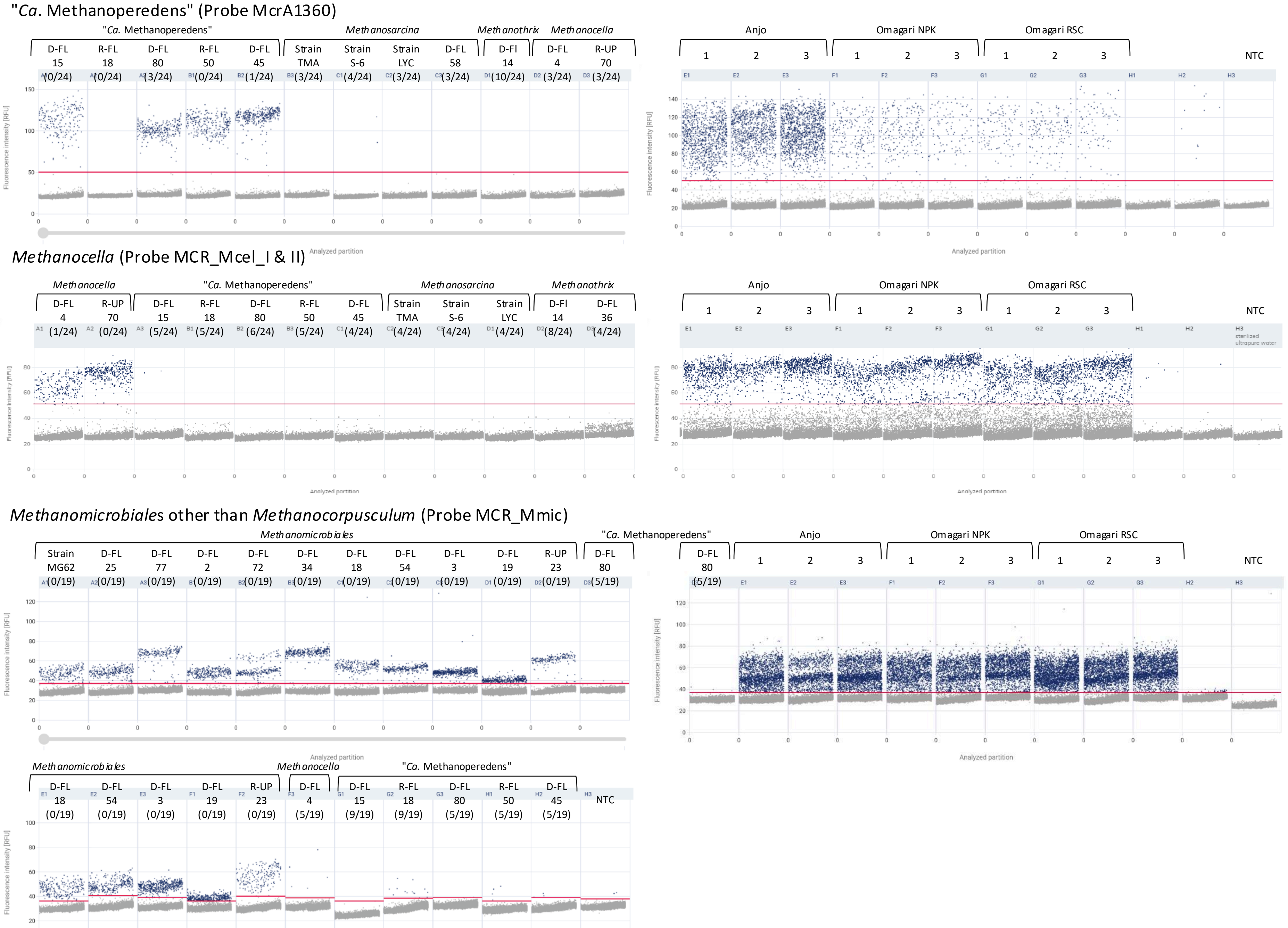

Fig. S1. Continued.
